# Positive Magnetoresistance and Chiral Anomaly in Exfoliated Type-II Weyl Semimetal *T*_d_-WTe_2_

**DOI:** 10.3390/nano11102755

**Published:** 2021-10-18

**Authors:** Rajdeep Adhikari, Soma Adhikari, Bogdan Faina, Marc Terschanski, Sophie Bork, Claudia Leimhofer, Mirko Cinchetti, Alberta Bonanni

**Affiliations:** 1 Institut für Halbleiter-und-Festkörperphysik, Johannes Kepler University, Altenbergerstr. 69, A-4040 Linz, Austria; soma.adhikari@jku.at (S.A.); Bogdan.Faina@jku.at (B.F.); 2 Department of Physics, TU Dortmund, Otto-Hahn-Straße 4, 44227 Dortmund, Germany; marc.terschanski@tu-dortmund.de (M.T.); sophie.bork@tu-dortmund.de (S.B.); mirko.cinchetti@tu-dortmund.de (M.C.); 3 Institut für Polymerwissenschaften, Johannes Kepler University, Altenbergerstr. 69, A-4040 Linz, Austria; claudia.leimhofer_1@jku.at

**Keywords:** Weyl semimetals, mechanical exfoliation, topological semimetals, positive magnetoresistance, chiral anomaly

## Abstract

Layered van der Waals semimetallic $T_{d}$-WTe${}_{2}$, exhibiting intriguing properties which include non-saturating extreme positive magnetoresistance (MR) and tunable chiral anomaly, has emerged as a model topological type-II Weyl semimetal system. Here, ∼45 nm thick mechanically exfoliated flakes of $T_{d}$-WTe${}_{2}$ are studied via atomic force microscopy, Raman spectroscopy, low-*T*/high-$\mu_{0}H$ magnetotransport measurements and optical reflectivity. The contribution of anisotropy of the Fermi liquid state to the origin of the large positive transverse ${\mathrm{MR}}_{⊥}$ and the signature of chiral anomaly of the type-II Weyl Fermions are reported. The samples are found to be stable in air and no oxidation or degradation of the electronic properties is observed. A transverse ${\mathrm{MR}}_{⊥}$∼1200 % and an average carrier mobility of 5000 cm${}^{2}$V${}^{-1}$s${}^{-1}$ at T=5K for an applied perpendicular field $\mu_{0}H_{⊥}=7\;T$ are established. The system follows a Fermi liquid model for T≤50K and the anisotropy of the Fermi surface is concluded to be at the origin of the observed positive MR. Optical reflectivity measurements confirm the anisotropy of the electronic behaviour. The relative orientation of the crystal axes and of the applied electric and magnetic fields is proven to determine the observed chiral anomaly in the in-plane magnetotransport. The observed chiral anomaly in the WTe${}_{2}$ flakes is found to persist up to T=120K, a temperature at least four times higher than the ones reported to date.

## 1. Introduction

The presence of accidental two-fold degeneracies in the electronic band structures of solids leads to linear energy dispersions in the vicinity of the energy-degenerate points or nodes [1,2,3]. The emerging low energy excitations near these degenerate points follow a photon-like linear dispersion and can be described by the Weyl equation [2,4]. The signatures of such low energy Weyl Fermion-like excitations in condensed matter systems were recently observed in bulk NbAs [5] and TaAs crystals [6,7]. These materials are symmetry-protected topological states of matter and are characterized by conduction and valence bands which join with a linear dispersion around a pair of Weyl nodes [2,3,5,6,8,9]. Like in high energy physics, in low energy condensed matter systems, the Dirac, Weyl and Majorana Fermions constitute the family of elementary Fermions [4,10,11] and are an appealing workbenck for future quantum technologies. The Weyl semimetals (WSM) are topological semimetals hosting Weyl quasiparticles (WQP) [2,3,12]. The WQPs are massless spin-1/2 Fermions, but their dispersion resembles the one of photons, due to the effective relativistic symmetry and the gapless Weyl nodes. The momenta *k* of the WQPs are projected either parallel or antiparallel to their spins and are distinguished through the quantum number chirality χ. The quantum expectation value of the chiral current in WSM is not conserved [2,9,12], leading to non-conservation of the chiral current, also known as chiral anomaly or Adler–Bell–Jakiw (ABJ) anomaly [13,14,15,16,17]. In condensed matter physics, the WSM are either of type-I (WSM-I) or of type-II (WSM-II). The WSM-II are known to break the Lorentz symmetry, which is-in contrast- conserved in the type-I WSMs [2]. The violation of the Lorentz symmetry leads to a tilted Weyl cone in the momentum space and makes these materials compelling for the study of exotic Lorentz violation theories that are beyond the Standard Model [3]. The tilted Weyl cone combined with the broken chiral symmetry results in the onset of quantum mechanical and topological mechanisms, such as intrinsic anomalous Hall effects [18], Klein tunneling [19], Landau level collapse [20] and ABJ anomaly [13,16,17], both in WSM-I and in WSM-II.

The presence of tilted Weyl cones and of low energy excitations violating the Lorentz invariance in the vicinity of the Weyl points were predicted for the electronic band structure of WTe${}_{2}$, a transition metal dichalcogenide (TMDC) semimetal with layered van der Waals structure [21,22]. In addition to the presence of the Fermi arc at the Fermi surface [23,24,25], anisotropic transport properties including extreme transverse magnetoresistance and the anisotropic ABJ anomaly, are reported for both bulk and few layers WTe${}_{2}$ flakes [15,26,27,28,29,30,31,32,33,34,35,36]. The tilted Weyl cone was also observed in the TMDC semimetal MoTe${}_{2}$ [37,38] and in LaAlGe [39]. Another characteristic feature of WTe${}_{2}$ is the extreme positive transverse magnetoresistance ${\mathrm{MR}}_{⊥}$, which can reach values as high as $10^{5}\%$ for magnetic fields ∼9 T applied parallel to the c−axis of bulk or thin flakes of WTe${}_{2}$ in the Fermi liquid phase at T∼ 100 mK [40,41,42]. In addition, evidence of topologically protected conducting edge states [43] and flat-band superconductivity in close proximity to Pd [44] and Nb [45] was shown in bulk and few layers WTe${}_{2}$ flakes, while quantum spin Hall states are found in mechanically exfoliated monolayer WTe${}_{2}$[46]. Most of the reported literature on $T_{d}$-WTe${}_{2}$ concerns bulk crystals or mechanically exfoliated flakes. While the bulk crystals are chemically stable [40], the ultrathin exfoliated flakes of $T_{d}$-WTe${}_{2}$ are reported to be prone to oxidation and require inert ambient for fabrication and sample processing [43]. In particular, it is not evident whether the crystal exfoliates along a preferred direction corresponding to the a− or b−axes [14]. In addition, apart from the observed extreme positive ${\mathrm{MR}}_{⊥}$, weak-antilocalization (WAL) and negative longitudinal magnetoresistance ${\mathrm{MR}}_{\|}$ with chiral anomaly are reported in exfoliated flakes of $T_{d}$-WTe${}_{2}$ [14,34].

Here, mechanically exfoliated ∼45 nm thin flakes of $T_{d}$-WTe${}_{2}$ are studied via atomic force microscopy (AFM), Raman spectroscopy, optical reflectivity and low-*T*/high-$\mu_{0}H$ magnetotransport measurements. Beside confirming the chemical stability of the system in ambient conditions, a Fermi liquid behavior is found, with large positive ${\mathrm{MR}}_{⊥}$ up to ∼1200%, average carrier mobility $\mu_{\mathrm{av}}$∼5000 cm${}^{2}$V${}^{-1}$s${}^{-1}$ and chiral anomaly persisting up to T∼ 120 K.

## 2. Materials and Methods

The WTe${}_{2}$ flakes are fabricated via mechanical exfoliation from a bulk (mother) crystal obtained commercially from hqgraphene (http://www.hqgraphene.com (accessed on 1 September 2019). Micromechanical cleavage is repeated using fresh Nitto tapes until ∼45 nm thin flakes are obtained. Upon exfoliation, the flakes are transferred onto Gelpack Grade 4 viscoelastic polydimethylsiloxane (PDMS) stamps with a rigid metallic support. The mechanical exfoliation process results in an ensemble of flakes with diverse sizes, geometries and thicknesses, distributed over the PDMS stamp. The exfoliated flakes on the PDMS are then analysed for thickness and number of layers using a high resolution Keyence VHX-7000 optical microscope (Keyence, Osaka, Japan) operated in transmission mode. Flakes of uniform thickness are then transferred onto prepatterned SiO${}_{2}$/$p^{++}$-Si substrates with markers and metal contact pads. The flakes transferred onto the pristine substrates are used for AFM, Raman spectroscopy and optical measurements, while the samples fabricated on the prepatterned substrates are employed to study the electronic properties of the exfoliated WTe${}_{2}$ flakes by low-*T*/high-$\mu_{0}H$ magnetotransport measurements.

The $\left(1\times 1\right)$ cm${}^{2}$ SiO${}_{2}$/$p^{++}$-Si substrates with a SiO${}_{2}$ thickness of 90 nm are spin coated with S1805 positive photoresist followed by soft baking at 90 ${}^{\circ }$C. A Süss Mask aligner photolithography system is employed to expose the S1805 coated substrates to an ultraviolet mercury lamp through a window mask. The substrates are developed using a conventional MS-519 photolithography developer and subsequently transferred into a sputtering chamber for the deposition of 10 nm thick Pt contacts. The metallic contact pads in van der Pauw 4-probe geometry are then obtained by rinsing away the photoresist with warm acetone for 15 seconds in an ultrasonic bath.

The exfoliated WTe${}_{2}$ flakes are transferred onto both pristine and prepatterned substrates using an indigeneously developed viscoelastic dry transfer system [47]. Gold wires with a diameter of 25 μm are bonded to the Pt contact pads using In as conducting adhesive. It is worth noting, that the mechanical exfoliation and the viscoelastic dry transfer are carried out in air ambient. The stability of the samples in ambient conditions is discussed in the next section. This is in contrast to what generally reported in literature, where the fabrication and transfer of the flakes takes place in vacuum or in inert atmosphere [28,43]. The entire procedure of sample fabrication and including the mechanical exfoliation, the viscoelastic dry transfer of the WTe${}_{2}$ flakes on prepatterned Pt contacts, the Au wire bonding and the transfer of the samples into the cryostat for measurements is achieved in less than 30 min, thereby ensuring minimal oxidation of the flakes. This fabrication protocol minimizes the detrimental effects of surface oxidation on the electronic properties of the WSM-II WTe${}_{2}$ [48,49].

**Figure 1:**
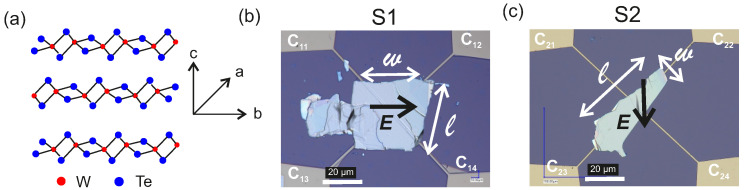
(**a**): Schematic illustration of the crystal structure of $T_{d}$-WTe${}_{2}$ showing the directions of the *a*−, b−, and c−axes. (**b**,**c**): Optical image of the WTe${}_{2}$ samples S1 and S2, respectively.

A schematic representation of the semimetallic bulk crystal structure of $T_{d}$-WTe${}_{2}$ highlighting the a−, b− and c− directions of the space group $C_{2v}^{7}$ ($Pmn2_{1}$) distorted orthorhombic basis, is shown in Figure 1a. Each unit cell is composed of two W and four Te atoms and the W-Te bond lengths vary between 2.7Å and 2.8Å [40,50]. The bulk WTe${}_{2}$ exhibits a $T_{d}$ stacking order in which the atoms in the upper layer are rotated by 180 ${}^{\circ }$ w.r.t. the atoms in the lower layer, as sketched in Figure 1a. In the $T_{d}$-WTe${}_{2}$, the *a*-axis is populated by the W-chain while the axis *b* is orthogonal to it. The c−axis is perpendicular to the ab− plane. Two exemplary samples are considered, namely:

(i) Sample S1: in the van der Pauw geometry the excitation current $I_{\mathrm{ac}}$ is applied between the contacts $C_{11}$ and $C_{12}$ while the resulting voltage $V_{\mathrm{xx}}$ is measured across $C_{13}$ and $C_{14}$, so that the electric field *E* due to $I_{\mathrm{ac}}$ is aligned exactly along *w*, as visualized in the optical microscopy image in Figure 1b;

(ii) Sample S2: in the van der Pauw geometry $I_{\mathrm{ac}}$ is applied between the contacts $C_{21}$ and $C_{22}$ while $V_{\mathrm{xx}}$ is measured across $C_{23}$ and $C_{24}$, so that *E* is slightly misaligned with respect to *w*, as evidenced in the optical microscopy image in Figure 1c.

The length and width of the rectangle-like flakes are indicated by *l* and *w*, respectively. In the absence of a conclusive evidence for the precise orientations of the a− and b−axes, the geometry of the studied flakes is described by *l* and *w*, while the c−axis is the one perpendicular to the plane of the flakes.

## 3. Results and Discussions

### 3.1. Atomic Force Microscopy and Raman Spectroscopy

**Figure 2:**
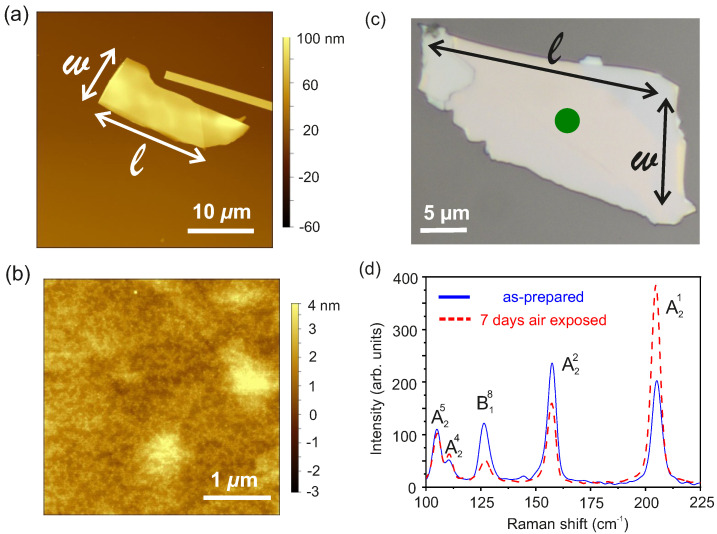
(**a**): AFM image of an exfoliated WTe${}_{2}$ flake dry transferred onto the SiO${}_{2}$/$p^{++}$-Si substrate. (**b**): Surface morphology recorded for a $\left(3.5\times 3.5\right)\;\mu m^{2}$ AFM scan area of the transferred WTe${}_{2}$ flake. (**c**): Optical microscopic image of 45 nm thick WTe${}_{2}$ flake used to measure Raman spectroscopy. The dot indicates the position of the laser spot during the Raman measurements. (**d**): Raman spectra collected from the specimen in (**c**) as-prepared and after seven days of exposure to air ambient.

The surface morphology and the thickness of the WTe${}_{2}$ flakes are measured using a VEECO Dimension 3100 AFM system. The AFM image of a WTe${}_{2}$ flake transferred onto a SiO${}_{2}$/$p^{++}$-Si substrate is provided in Figure 2a. The height profile of the flake is analysed using the Gwiddyon data analysis software and a thickness of 45 nm is determined. The surface morphology of the flake is shown in Figure 2b and a root mean square surface roughness of ∼0.45 nm is estimated.

The chemical stability and crystallographic phase of the WTe${}_{2}$ flakes are studied using Raman spectroscopy carried out in a WIRec Alpha 300 R-Raman-System with a double frequency Nd:YAG laser of wavelength 532 nm. The objective allows a laser beam spot diameter of ∼2 μm. The samples are positioned on a XY-translation stage and a camera system enables guiding the sample in the laser spot. A total of 33 Raman vibrations are predicted by group theory [51] and the irreducible representation of the optical phonons at the Γ point of the Brilloiun zone of the bulk $T_{d}-$WTe${}_{2}$ is given by:

(1) $$\Gamma_{\mathrm{bulk}}=11A_{1}+6A_{2}+11B_{1}+5B_{2}$$

where $A_{1}$, $A_{2}$, $B_{1}$ and $B_{2}$ are Raman active phonon modes. In this work, the Raman modes have been excited along the c−axis of the $T_{d}-$WTe${}_{2}$ crystal, i.e., the laser beam is directed perpendicular to the plane of the WTe${}_{2}$ flake and of the substrate. Since the Raman excitations reported here are unpolarized, neither the incident, nor the scattered electric field vectors $\vec{e_{i}}$ and $\vec{e_{s}}$ are aligned along the a− or c−axes. An optical microscopy image of the flake studied by Raman spectroscopy is shown in Figure 2c, while the room temperature Raman spectra recorded for the as-prepared and air aged WTe${}_{2}$ flake are given in Figure 2d. A total of five Raman active modes with peaks centered at 105.0 cm${}^{-1}$, 110.5 cm${}^{-1}$, 126.5 cm${}^{-1}$, 157.2 cm${}^{-1}$, and 205.3 cm${}^{-1}$ have been recorded. The Raman active modes are labelled as $A_{q}^{p}$ and $B_{q}^{p}$, where q={1,2} and p∈Z to uniquely identify the Raman mode. Here, the five detected Raman peaks are assigned to the $A_{2}^{5}$, $A_{2}^{4}$, $B_{1}^{8}$, $A_{2}^{2}$, and $A_{2}^{1}$ allowed Raman active modes [50,51]. Upon comparison with the calculated resonances, it is found that the experimental $A_{2}^{4}$ is blue shifted, while the other four Raman active modes are red shifted due to the stress built up when the flake is transferred onto the rigid substrate. Upon measurement, the sample has been exposed to air for seven days and then Raman spectra have been recorded. The Raman spectrum for the air aged sample, represented by the dashed curve in Figure 2d is found to show the allowed Raman modes of the optical phonons as recorded for the pristine flake. No peaks related to oxides of Te or W are detected, confirming the chemical stability of the WTe${}_{2}$ flakes.

### 3.2. Out-of-Plane Magnetotransport

**Figure 3:**
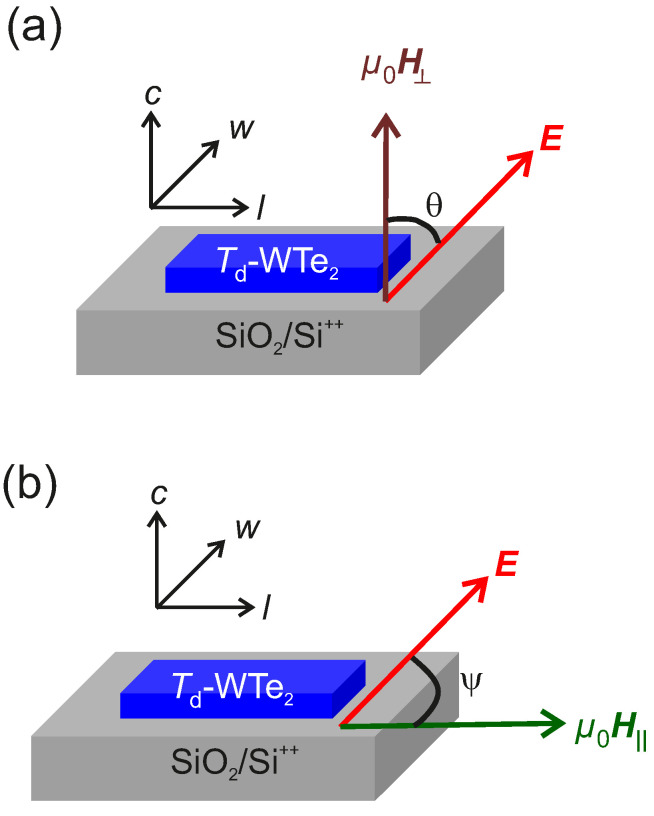
Schematic illustration of the relative orientations of *E*, $\mu_{0}H$, *l* and *w* relevant for the measurements of (**a**): ${\mathrm{MR}}_{⊥}$ and (**b**): ${\mathrm{MR}}_{\|}$.

Low-*T*/high-$\mu_{0}H$ magnetotransport measurements are carried out on the samples S1 and S2 in a Janis Super Variable Temperature 7TM-SVM cryostat (Janis Cryogenics, Westerville, OH, USA) equipped with a 7 T superconducting magnet. Prior to the measurements, the Ohmic nature of the Pt contacts to the WTe${}_{2}$ flakes is confirmed by the linear I−V characteristics measured with a high precision Keithley 4200 SCS dc source-measure unit (SMU). The longitudinal resistance $R_{\mathrm{xx}}$ as a function of *T* and $\mu_{0}H$ has been measured by employing a lock-in amplifier (LIA) ac technique. The $I_{\mathrm{ac}}$ is sourced from a Standord Research SR830 LIA, while the generated voltage $V_{\mathrm{xx}}$ is measured in a phase locked mode as a function of *T* and $\mu_{0}H$. The applied current is limited to 10 μA to minimize Joule heating and subsequent thermogalvanic effects due to the constraints imposed by the low dimensionality of the samples. The lock-in expand function is employed to enhance the sensitivity of the LIA. All measurements have been performed at a frequency of 127 Hz. The chosen reference axes for the applied $\mu_{0}H$, for *E* due to $I_{\mathrm{ac}}$, and for the *l* and *w* dimensions of the specimens - identified to characterize the transverse magnetoresistance ${\mathrm{MR}}_{⊥}$ and the longitudinal magnetoresistance ${\mathrm{MR}}_{\|}$-are shown in Figure 3a,b, respectively. In Figure 3a, θ is the angle between $\mu_{0}H_{⊥}$ applied along the c−axis and the ab−plane, while *E* is oriented along *w*. The $\mu_{0}H_{⊥}$ has been recorded for $\theta =0^{\circ }$ and $90^{\circ }$. The ${\mathrm{MR}}_{\|}$ for S1 and S2 are measured by applying an in-plane magnetic field $\mu_{0}H_{\|}$ at an angle ψ w.r.t. *E*, while *E* is always applied along *w*. Thus, there are two possible configurations of the relative orientations of *E*, $\mu_{0}H_{\|}$ w.r.t. *w*, namely: (i) $\left[\mu_{0}H_{\|}\;\|\;\left(E\;\|\;w\right)\right]$ and (ii) $\left[\mu_{0}H_{\|}\;⊥\;\left(E\;\|\;w\right)\right]$.

**Figure 4:**
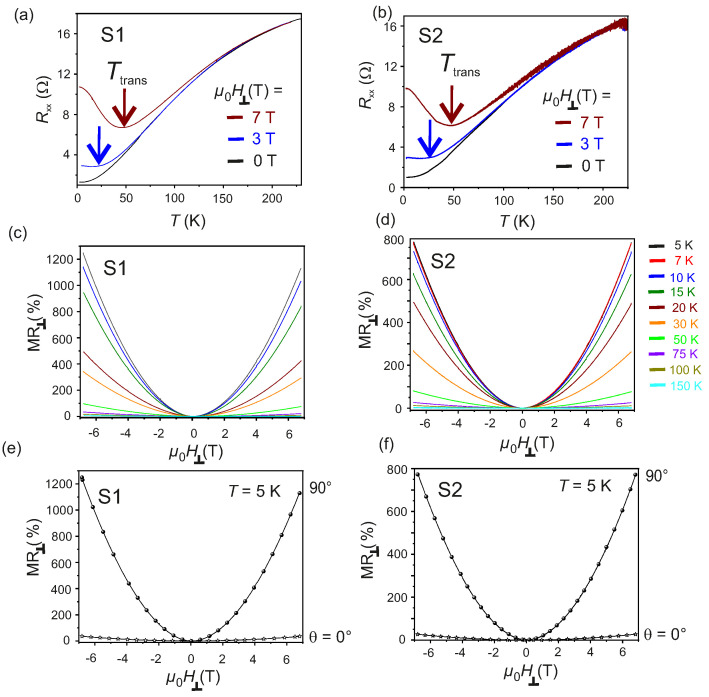
(**a**,**b**): $R_{\mathrm{xx}}$ as a function of *T* at $\mu_{0}H_{⊥}=0\;T$, $\mu_{0}H_{⊥}=3\;T$ and $\mu_{0}H_{⊥}=7\;T$ for S1 and S2, respectively. (**c**,**d**): ${\mathrm{MR}}_{⊥}$ as a function of $\mu_{0}H_{⊥}$ measured in the range 5K≤T≤150K for S1 and S2, respectively. (**e**,**f**): ${\mathrm{MR}}_{⊥}$ as a function of $\mu_{0}H$ at $\theta =0^{\circ }$ and $\theta =90^{\circ }$ at T=5K for S1 and S2, respectively.

The evolution of longitudinal resistance $R_{\mathrm{xx}}$ as a function of *T* in the interval 5K≤T≤225K for S1 and for S2 is given in Figure 4a,b, respectively. The $R_{\mathrm{xx}}$–*T* behavior is studied while the samples are cooled down, both for $\mu_{0}H_{⊥}=0$ and for $\mu_{0}H_{⊥}\neq 0$ and the measurements are referred to as zero field cooled (ZFC) and field cooled (FC), respectively. The FC $R_{\mathrm{xx}}$ are measured by applying $\mu_{0}H_{⊥}=$ 3 T and 7 T, as shown in Figure 4a,b. The monotonous decrease of $R_{\mathrm{xx}}$ with decreasing *T* for $\mu_{0}H_{⊥}=0$ is a signature of the metallic behavior of the exfoliated $T_{d}$-WTe${}_{2}$ flakes. For $\mu_{0}H_{⊥}\geq 3\;T$, $R_{\mathrm{xx}}$ essentially follows the ZFC behavior down to $T_{\mathrm{Trans}}$. At $T=T_{\mathrm{Trans}}$—indicated by the arrows (↓) in Figure 4a,b—a transition in the electronic phase of the flakes from metallic to insulating is found for both S1 and S2. This behavior is consistent with the one previously observed for bulk and thin $T_{d}$-WTe${}_{2}$ layers [35,40,42,52,53]. It is also noted, that the magnitude of the $\mu_{0}H_{⊥}$- induced change in $R_{\mathrm{xx}}$ increases with decreasing *T* and with increasing $\mu_{0}H_{⊥}$. The magnetoresistance, defined as:

(2) $$\mathrm{MR}=\frac{R_{\mathrm{xx}}\left(\mu_{0}H\right)-R_{\mathrm{xx}}\left(0\right)}{R_{\mathrm{xx}}\left(0\right)}\times 100\%$$

is a fingerprint of the microscopic physical mechanism governing the electronic properties of any trivial or non-trivial electronic system. Here, $R_{\mathrm{xx}}\left(\mu_{0}H\right)$ and $R_{\mathrm{xx}}\left(0\right)$ are the resistances of the system under an applied field $\mu_{0}H$ and in zero field, respectively. The ${\mathrm{MR}}_{⊥}$ of the two samples S1 and S2 are estimated as a function of an applied field $\mu_{0}H_{⊥}$ at different *T* in the range 5K≤T≤150K and are reported in Figure 4c,d. Large positive ${\mathrm{MR}}_{⊥}$∼1200% and ∼800% are found at T=5K, under the maximum applied field $\mu_{0}H_{⊥}=\;7\;T$ for S1 and S2, respectively. The estimated ${\mathrm{MR}}_{⊥}$ for both samples follow the power-law behavior $\mathrm{MR}∼mH^{n}$ [42,54], where m is a proportionality constant and n∈R is the power index. The value of *n* is estimated by numerical fitting of the ${\mathrm{MR}}_{⊥}$ behavior for both S1 and S2 as a function of $\mu_{0}H_{⊥}$ and is found to be 1.9≤n≤2.1 in the range 5K≤T≤150K [35,40,41,42]. It is noted, that no Shubnikov-de Haas (SdH) oscillations have been observed in the ${\mathrm{MR}}_{⊥}$ of both samples, even up to the maximum $\mu_{0}H_{⊥}$. The ${\mathrm{MR}}_{⊥}$ as a function of $\mu_{0}H_{⊥}$ for $\theta =0^{\circ }\;\mathrm{and}\;90^{\circ }$ and T=5K are also recorded for S1 and S2 and reported in Figure 4e,f, respectively. An anisotropic behavior of ${\mathrm{MR}}_{⊥}$ is observed for the $T_{d}$-WTe${}_{2}$ flakes studied here [7,15,42,53].

**Figure 5:**
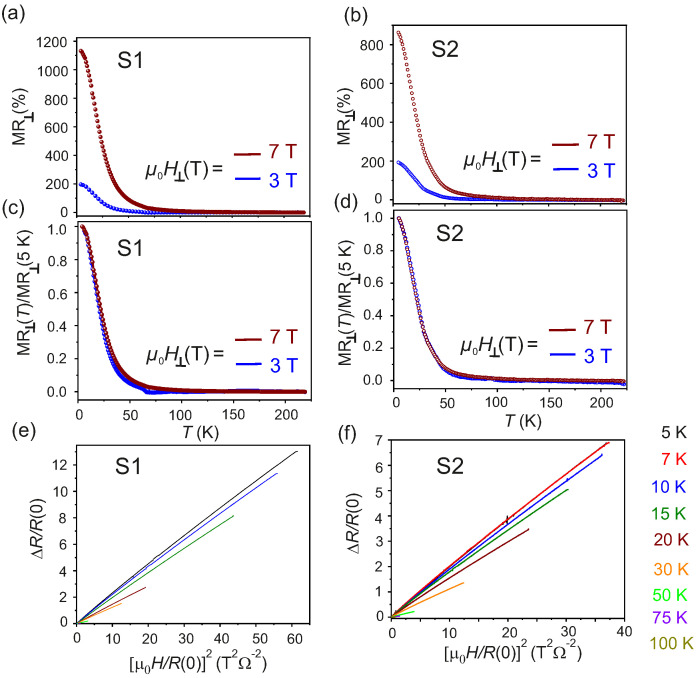
(**a**,**b**): FC transverse MR as a function of *T* recorded for $\mu_{0}H_{⊥}=3\;T$ and 7T for samples S1 and S2, respectively. (**c**,**d**): Estimated normalized ${\mathrm{MR}}_{⊥}$ defined as the ratio of ${\mathrm{MR}}_{⊥}\left(T\right)$ to ${\mathrm{MR}}_{⊥}\left(5\;K\right)$ recorded by applying $\mu_{0}H_{⊥}=3\;T$ and $\mu_{0}H_{⊥}=7\;T$ for S1 and S2, respectively. (**e**,**f**): calculated Kohler’s plots of S1 and S2 in the range 5K≤T≤100K.

The evolution of ${\mathrm{MR}}_{⊥}$ as a function of *T* for $\mu_{0}H_{⊥}=3\;T$ and $\mu_{0}H_{⊥}=7\;T$ is shown in Figure 5a,b for S1 and S2, respectively. The positive ${\mathrm{MR}}_{⊥}$ sets in for T≤75K and for a critical field $\mu_{0}H_{c}\geq 3\;T$, in accord with the $R_{\mathrm{xx}}$-*T* behavior previously discussed. A relevant feature of the observed large ${\mathrm{MR}}_{⊥}$, is the presence of a magnetic field-dependent critical turn-on temperature $T_{\mathrm{Trans}}$ for $\mu_{0}H_{⊥}\geq 3\;T$. However, such critical temperature is absent when the samples are cooled down in the presence of the field $\mu_{0}H_{\|}$. The transition from metallic to insulating state is observed in several other material systems exhibiting colossal positive MR, where the extreme magnitude of the MR is attributed to a magnetic field-driven metal-to-insulator (MIT) transition [55,56,57], due to a field-induced excitonic gap in the linear spectrum of the Coulomb interacting quasiparticles, leading to an excitonic insulator phase [42,58]. The excitonic gap is expected to follow the relation: $\Delta_{T}\left(\mu_{0}H-\mu_{0}H_{c}\right)\to {\left[\mu_{0}H-\mu_{0}H_{c}\left(T\right)\right]}^{\frac{1}{2}}$, where $\mu_{0}H_{c}$ is the threshold magnetic field and the dependence of the excitonic gap on the magnetic field is characteristic of a second order phase transition.

The normalized ${\mathrm{MR}}_{⊥}$ for S1 and S2, defined as the ratio between ${\mathrm{MR}}_{⊥}$ measured at any *T* (${\mathrm{MR}}_{⊥}\left(T\right)$) and ${\mathrm{MR}}_{⊥}$ at T=5K (${\mathrm{MR}}_{⊥}\left(5\;K\right)$), are plotted as a function of *T* for $\mu_{0}H_{⊥}=3\;T$ and $\mu_{0}H_{⊥}=7\;T$ in Figure 5c,d, respectively. It is observed, that the ${\mathrm{MR}}_{⊥}$ have the same *T*-dependence for both S1 and S2 WTe${}_{2}$ flakes, as inferred from the collapse of the two curves. This behavior of the normalized ${\mathrm{MR}}_{⊥}$ is inconsistent with the existence of a $\mu_{0}H$-dependent excitonic gap [42,53,58]. It is, thus, concluded that the origin of the MIT observed here in the $R_{\mathrm{xx}}$–*T* behavior for $\mu_{0}H_{⊥}\geq 3\;T$, lies in the evolution of the electronic structure of $T_{d}$-WTe${}_{2}$. From angle-dependent photoemission spectroscopic studies on bulk $T_{d}$-WTe${}_{2}$, it was shown that the presence of minute electron and hole pockets of equal size at low *T* is responsible for the remarkably large positive ${\mathrm{MR}}_{⊥}$, due to a *T*-dependent charge compensation mechanism [25,59]. The anisotropic behaviour of the Fermi surface of $T_{d}$-WTe${}_{2}$ is reflected in an anisotropic ${\mathrm{MR}}_{⊥}$ as a function of the direction of $\mu_{0}H$ defined by θ, which is also observed in the $T_{d}$-WTe${}_{2}$ flakes measured in this work, as evidenced in Figure 5e,f. Similar results are obtained when a 45 nm thick $T_{d}$-WTe${}_{2}$ flake is contacted with Au (instead of Pt) and the results are shown in Figures S4 and S5 of the Supplementary Materials. Therefore, it can be concluded, that the transverse magnetotransport properties of mechanically exfoliated $T_{d}$-WTe${}_{2}$ are independent of the metal employed to contact the semimetallic flakes and also of the exposure of the flakes to air ambient.

The presence of the electron and hole pockets in the elctronic bands of $T_{d}$-WTe${}_{2}$ is probed by a Kohler’s analysis of the $R_{\mathrm{xx}}$–$\mu_{0}H_{⊥}$ curves at different *T*. According to the Kohler’s theory, the change in the isothermal longitudinal resistance $R_{\mathrm{xx}}$ for a conventional metal in an applied field $\mu_{0}H$ obeys the functional relation:

(3) $$\frac{\Delta R_{\mathrm{xx}}}{R(0)}=F\left(\frac{\mu_{0}H}{R(0)}\right)$$

where R(0) is the ZFC resistance at *T*. The Kohler’s behavior is due to the fact, that conventional metals host a single kind of charge carriers. In a weak field limit, the MR of most metals follows a quadratic dependence on $\mu_{0}H$, i.e., $\mathrm{MR}\propto \left(\alpha +\beta \mu_{0}H^{2}\right)$, with α and β proportionality constants [54]. The resistance $R\left(0\right)\;\mathrm{is}\;\propto \frac{1}{\tau }$, where τ is the scattering time of the itinerant charge carriers in a metallic system. Therefore, a plot of $\frac{\Delta R_{\mathrm{xx}}}{R(0)}$ vs. ${\left(\frac{H}{R(0)}\right)}^{2}$ is expected to collapse into a single *T*-independent curve, provided that the number of charge carriers, the type of charge carriers, and the electronic disorder in the system remain constant over the measured *T* range. The Kohler’s plots for S1 and S2 measured at various *T* are reported in Figure 5e,f, respectively. A significant deviation is observed in the scaled transverse ${\mathrm{MR}}_{⊥}$. Due to spin dependent scattering, such deviations are common in magnetically doped topological systems [60], but in a non-magnetic system such as $T_{d}$-WTe${}_{2}$ this behavior indicates that the electronic bands contain both electrons and holes as charge carriers. Therefore, the formation of excitons leads to a change in the carrier density, resulting in the observed deviation from the Kohler’s rule in the thin flakes of $T_{d}$-WTe${}_{2}$ as observed previously in bulk systems [42,59]. However, as reported by Wang et al. [61], bulk $T_{d}$-WTe${}_{2}$ crystal grown by chemical vapour transport follows the Kohler’s rule, which is in contrast to the behavior observed here.

The values of the average carrier mobility $\mu_{\mathrm{av}}$, i.e., the mean value of the electron and hole mobilities, are calculated for both samples considered here, by fitting ${\mathrm{MR}}_{⊥}$ with the Lorentz law [41,54] according to the relation:

(4) $$\frac{\Delta R}{R\left(0\right)}=\left[1+{\left(\mu_{\mathrm{av}}\mu_{0}H\right)}^{2}\right]$$

**Figure 6:**
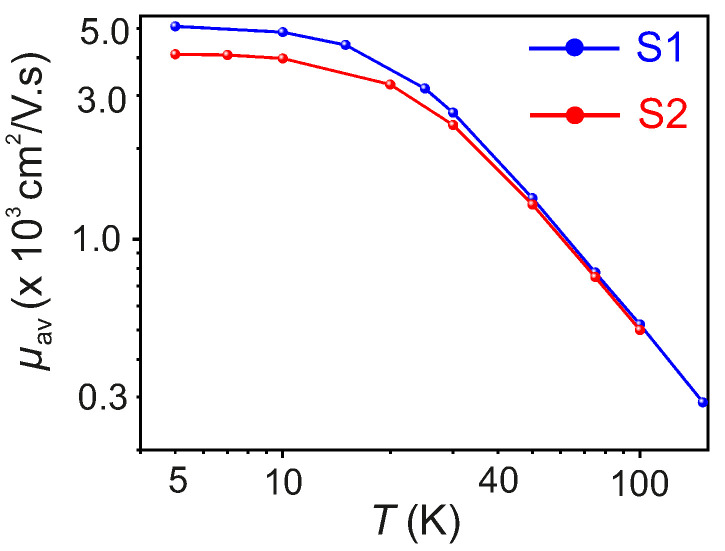
$\mu_{\mathrm{av}}$ as a function of *T* for samples S1 and S2.

The estimated $\mu_{\mathrm{av}}$ for S1 and S2 as a function of *T* are provided in Figure 6. Due to the dominant electron-phonon correlation, a monotonic decrease of $\mu_{\mathrm{av}}$ is observed for T>50K, both in S1 and S2. However, for T≤50K, the estimated values of $\mu_{\mathrm{av}}$ with decreasing *T* exhibit a plateau in the logarithmic scale with estimated $\mu_{\mathrm{av}}≃5000$ cm${}^{2}$V${}^{-1}$s${}^{-1}$ and $\mu_{\mathrm{av}}≃4000$ cm${}^{2}$V${}^{-1}$s${}^{-1}$ at T=5K for S1 and S2, respectively. The values of $\mu_{\mathrm{av}}$ obtained in this work are higher than those reported in literature for ultra-thin ∼9 nm flakes [35] measured at T=5K, but are comparable to those reported for bulk crystals of $T_{d}$-WTe${}_{2}$ grown with the Te-flux method [62]. The high carrier mobility in semimetallic $T_{d}$-WTe${}_{2}$ at low *T* points at a deviation from the electron-phonon coupling-dominated carrier transport.

**Figure 7:**
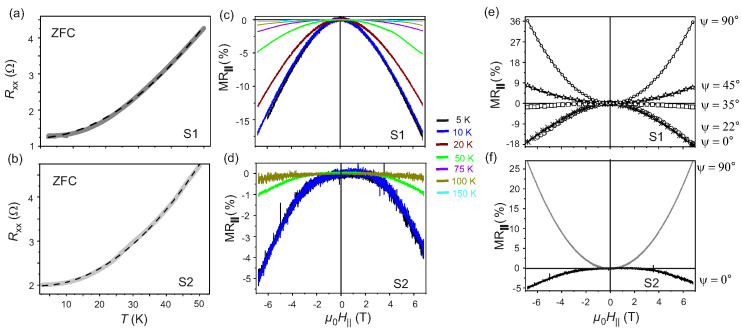
(**a**,**b**): $R_{\mathrm{xx}}$ as a function of *T* with $\mu_{0}H_{⊥}=0\;T$ for S1 and S2, respectively. (**c**,**d**): ${\mathrm{MR}}_{\|}$ recorded as a function of $\mu_{0}H_{\|}$ in the range 5K≤T≤100K for S1 and S2, respectively. (**e**,**f**): ${\mathrm{MR}}_{\|}$ as a function of the azimuthal angle ψ at T=5K for S1 and S2, respectively.

A deviation from linear behavior originating from the electron-phonon coupling at T≥50K is observed in the zero-field cooled $R_{\mathrm{xx}}$ as a function of *T* for both S1 and S2, as previously reported in Figure 4a,b, respectively. The behavior of $R_{\mathrm{xx}}$ as a function of *T* in the range 5K≤T≤50K is presented in Figure 7a,b for S1 and S2 and it follows the predictions of the Fermi liquid theory [42,54,59] with $R_{\mathrm{xx}}=\gamma_{0}+\gamma^{\prime }T^{2}$, where $\gamma_{0}$ and $\gamma^{\prime }$ are proportionality constants. For 5K≤T≤50K, electron-electron correlation is found to be the dominant mechanism in the Fermi liquid state of thin flakes of semimetallic $T_{d}$-WTe${}_{2}$ [25,42,59]. Thus, it is concluded, that the observed large positive ${\mathrm{MR}}_{⊥}$ occurs in the Fermi liquid state of the system, as endorsed by the observed anisotropic behavior of ${\mathrm{MR}}_{⊥}$, which is a signature of an anisotropic Fermi surface [25,40,41,42,59].

### 3.3. In-Plane Magnetotransport

The electronic band structure of WSM-II is characterized by the presence of an asymmetric electron dispersion responsible for the anisotropy in the electronic properties of these systems. In WSM-II, the breaking of the Lorentz invariance and of the chiral symmetry in massless Weyl Fermions under quantum fluctuations leads to the chiral anomaly, which is observed as a negative ${\mathrm{MR}}_{\|}$ under the condition of $\left(\mu_{0}H\;\|\;E\right)$. In particular, for the case of $T_{d}$-WTe${}_{2}$, for $\left[\mu_{0}H\;\|\;\left(E\;\|\;b\right)\right]$, the signature negative ${\mathrm{MR}}_{\|}$, anisotropic in the ab-plane of the orthorhombic lattice, is observed [14,34,52]. In the mechanically exfoliated flakes studied here, the directions of the a− and the b−axes are not determined apriori. As discussed above, the orientation of the studied flakes is therefore defined in terms of the dimensions *w* and *l*. Here, *E* is always parallel to *w*, while $\mu_{0}H_{\|}$ is applied at an azimuthal angle ψ w.r.t. $\left(E\;\|\;w\right)$. The angle ψ is varied between $0^{\circ }$ and $90^{\circ }$. The ${\mathrm{MR}}_{\|}$ has been estimated for both S1 and S2. The recorded ${\mathrm{MR}}_{\|}$ for S1 and S2 at T=5K are reported in Figure 7c,d, respectively. For S1, ${\mathrm{MR}}_{\|}$ is recorded for $\psi =0^{\circ },\;22^{\circ },\;35^{\circ },\;45^{\circ }\;\mathrm{and}\;90^{\circ }$. It is found, that the negative ${\mathrm{MR}}_{\|}$ due to the chiral anomaly disappears for $\psi ≳35^{\circ }$. For sample S2, ${\mathrm{MR}}_{\|}$ is collected for $\psi =0^{\circ },\;\mathrm{and}\;90^{\circ }$ solely to show the signature of anisotropy in ${\mathrm{MR}}_{\|}$ and the chiral anomaly. Negative ${\mathrm{MR}}_{\|}$ of magnitude −18% and −5% for $\mu_{0}H_{\|}=7\;T$ are observed at T=5K and $\psi =0^{\circ }$ for S1 and S2, respectively. The negative ${\mathrm{MR}}_{\|}$ for $\left[\mu_{0}H_{\|}\|\left(E\|w\right)\right]$ is a signature of chiral anomaly. The magnitude of ${\mathrm{MR}}_{\|}$ decreases with increasing *T* and vanishes at T≥150K, as shown in Figure 7c,d for S1 and S2, respectively.

The magnitude of the negative ${\mathrm{MR}}_{\|}$ is reduced on deviating from the parallel condition, i.e., for $\psi >0^{\circ }$. As depicted in Figure 7e, the magnitude of ${\mathrm{MR}}_{\|}$ estimated at $\psi =22^{\circ }$ is comparable to the one assessed for $\psi =0^{\circ }$. However, for $\psi =35^{\circ }$, a ${\mathrm{MR}}_{\|}∼$−1% is determined, which then reverses sign for increasing ψ and a positive ${\mathrm{MR}}_{\|}$ is measured for $\psi =45^{\circ }$ and $\psi =90^{\circ }$, respectively. Therefore, it is inferred that the assigned *w* axis of the flakes studied here is indeed aligned along the b−axis of the distorted rhombohedral unit cell of $T_{d}$-WTe${}_{2}$. Further, it is also established that the topological pumping of the chiral charge current in the WSM-II $T_{d}$-WTe${}_{2}$ occurs when $\mu_{0}H_{\|}$ is applied at angles $\psi <35^{\circ }$ w.r.t. E ‖ w. For $\left[\mu_{0}H_{\|}⊥\left(E\;\|\;w\right)\right]$, i.e., at $\psi =90^{\circ }$, a ${\mathrm{MR}}_{\|}=+36\%$ for $\mu_{0}H_{\|}=$ 7 T at T=5K is found, as shown in Figure 7e. Similar behavior is also observed for sample S2, as reported in Figure 7f. However, the reduced magnitude of ${\mathrm{MR}}_{\|}$ to −5% for $\psi =0^{\circ }$ indicates that for this flake, *w* is not aligned along the b−axis. The estimated values of ${\mathrm{MR}}_{⊥}$, ${\mathrm{MR}}_{\|}$, $\mu_{\mathrm{av}}$ at T=5K and the critical *T* up to which the chiral anomaly persists for samples S1 and S2 are summarized in Table 1.

**Figure 8:**
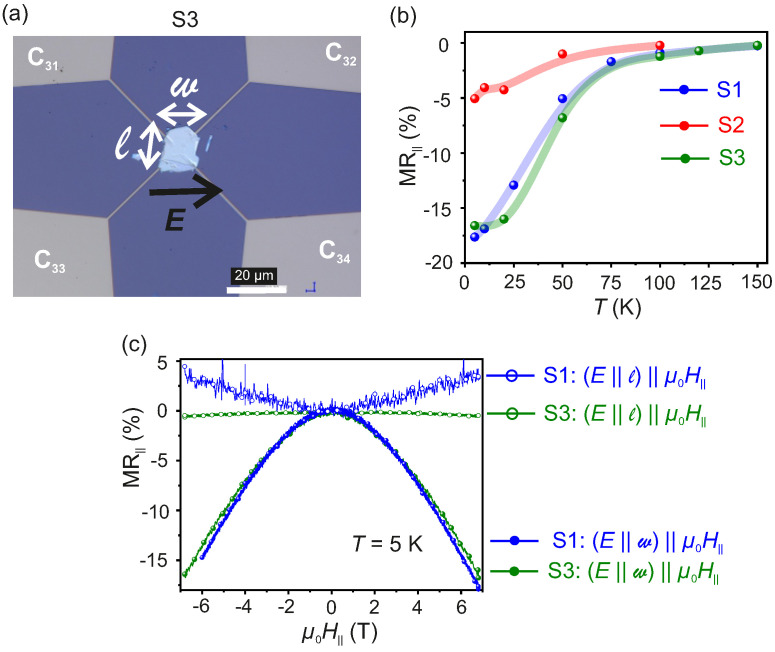
(**a**): Optical image of the sample S3. (**b**): Negative ${\mathrm{MR}}_{\|}$ as a function of $\mu_{0}H_{\|}$ — a fingerprint of the chiral anomaly measured for the configuration $\left[\mu_{0}H_{\|}\;\|\;\left(E\;\|\;w\right)\right]$ for samples S1, S2 and S3. (**c**): ${\mathrm{MR}}_{\|}$ measured at T=5K for samples S1 and S3 in the configurations $\left[\mu_{0}H_{\|}\;\|\;\left(E\|l\right)\right]$ and $\left[\mu_{0}H_{\|}\;\|\;\left(E\;\|\;w\right)\right]$.

To prove that $\left[\mu_{0}H_{\|}\;\|\;\left(E\;\|\;w\right)\right]$ is the necessary and sufficient condition to observe chiral anomaly in WTe${}_{2}$ and to confirm the reproducibility of the obtained results, a third sample S3 is fabricated following the methods described above and the ${\mathrm{MR}}_{\|}$ is measured as a function of *T*. For S3 in the van der Pauw geometry, $I_{\mathrm{ac}}$ is applied between the contacts $C_{31}$ and $C_{32}$ while $V_{\mathrm{xx}}$ is measured across $C_{33}$ and $C_{34}$, so that *E* is along *w*, as shown in the optical microscopy image given in Figure 8a. The value of ${\mathrm{MR}}_{\|}$ measured for $\mu_{0}H_{\|}=7\;T$ as a function of *T* is reported in Figure 8b for S1, S2 and S3. The measured ${\mathrm{MR}}_{\|}$ persists up to T≃120K for both S1 and S3, while for S2 the negative ${\mathrm{MR}}_{\|}$ survives up to T=100K. As seen in Figure 8b, the magnitude of ${\mathrm{MR}}_{\|}$ and its dependence on *T* are identical for S1 and S3, while the magnitude of ${\mathrm{MR}}_{\|}$ for S2 is reduced. This is due to the slight misalignment of the w−axis w.r.t. $I_{\mathrm{ac}}$ in S2. The critical *T* below which chiral anomaly can be observed in thin flakes of WTe${}_{2}$ reported in this work is at least four times higher than the one reported by Li et al. [34] who observed chiral anomaly up to 30 K. In contrast to those reported in literature [34], the WTe${}_{2}$ flakes used in this work are not exposed to any chemical or e-beam irradiation during the sample fabrication process. The pristine nature of the flakes used in this work is taken to be the reason for the observed critical temperature of 120 K. Additionally, the magnitude of ${\mathrm{MR}}_{\|}∼18\%$ measured at T=5K and $\mu_{0}H_{\|}=7\;T$ for S3 is one order of magnitude higher than the one already reported [34,63].

The observations of negative ${\mathrm{MR}}_{\|}$ or negative longitudinal magnetoresistance (NLMR) in bulk crystals of topological semimetals were shown to be dominated by current jetting [64,65,66,67,68,69]. Similar NLMR are found in bulk crystals of silver chalcogenides such as Ag${}_{2}$Se and Ag${}_{2}$Te with non-stochiometry as low as 1 part in 10${}^{4}$. Here, the fluctuations in conductivity due to the inhomogeneities in the ion distributions and the application of high magnetic fields of a few T applied parallel to the current, is inferred to be at the origin of the NLMR [70,71]. Current jetting effects in topological semimetals are shown to depend on carrier mobility, on the mutual directions of the applied electric and magnetic fields and on the strength of the applied magnetic field. In the presence of a strong $\mu_{0}H_{\|}$, the Weyl states in both WSM-I and WSM-II are quantized into Landau levels with the unique feature of the lowest Landau level (LLL) being chiral. The chiral charge density is given by $\rho^{5}=\left(N_{+}-N_{-}\right)/V$, where *V* is the sample volume and $N_{+}$ and $N_{-}$ are the number of right handed and left handed chiral Weyl Fermions, respectively. The quantity $\rho^{5}$ is conserved until $E\;\|\;\mu_{0}H_{\|}$. In terms of quantum field theory, $\nabla .J^{5}+\partial_{t}\rho^{5}=eC$ where $J^{5}$ is the chiral current density and C is the anomaly term given by $C=\left(e^{2}/4\pi^{2}\hbar^{2}\right)E.\mu_{0}H$. The conservation of $\rho^{5}$ is violated due to a non-zero C and the axial current is detected via negative ${\mathrm{MR}}_{\|}$. Therefore, the chiral anomaly induced negative ${\mathrm{MR}}_{\|}$ is a purely quantum effect involving the LLL and is observed on application of non-zero magnetic fields. On the other hand, in order to induce current jetting, the lower limit of the magnetic field $\mu_{0}H_{\mathrm{cj}}$ is given by the relation:

(5) $$\mu_{0}H_{\mathrm{cj}}=\frac{C}{\mu_{\mathrm{av}}}$$

where $\mu_{\mathrm{av}}$ is the average carrier mobility. The value of C is estimated to be ∼(5–10) for topological semimetals [67]. From the measured $\mu_{\mathrm{av}}≃5000$ cm${}^{2}$/V.s at T=5K of the samples studied in this work, the critical value of $\mu_{0}H_{\mathrm{cj}}$ is calculated to be ∼10 T, which largely exceeds the maximum $\mu_{0}H_{\|}=7\;T$ used in this work. This is in contrast to the $\mu_{0}H_{\mathrm{cj}}$ reported for bulk crystals of WSM-I where the carrier mobilities were found to be as high as $\left(5\times 10^{6}\right)$ cm${}^{2}$/V.s at T=5K, leading to $\mu_{0}H_{\mathrm{cj}}\leq$ 1 T [64,67]. Additionally, the flakes studied here have a thickness of 45 nm, which is more than four orders of magnitude lower than the one of bulk samples with dominant current jetting. Furthermore, the negative ${\mathrm{MR}}_{\|}$ is observed only under the condition $\left[\mu_{0}H_{\|}\;\|\;\left(E\;\|\;w\right)\right]$ for all the studied samples. No negative ${\mathrm{MR}}_{\|}$ is recorded in S1, S2 and S3 for the condition $\left[\mu_{0}H_{\|}\;\|\;\left(E\;\|\;l\right)\right]$, ruling out the geometric current jetting mechanism. The ${\mathrm{MR}}_{\|}$ for S1 and S3 measured at T=5K for both configurations, namely $\left[\mu_{0}H_{\|}\;\|\;\left(E\;\|\;l\right)\right]$ and $\left[\mu_{0}H_{\|}\;\|\;\left(E\;\|\;w\right)\right]$ are presented in Figure 8c. The obtained results indicate that the chiral anomaly can be observed only for a unique combination of the relative directions of *E*, $\mu_{0}H_{\|}$ and *w*. In addition, as shown in Figure S6a,b in the Supplementary Materials, only a positive ${\mathrm{MR}}_{\|}$ is measured for sample S4, where the flake is placed on the prefabricated contacts in such a way, that the *w* and *l* axes are misaligned w.r.t. the current and voltage leads with $\left(\mu_{0}H\;\|\;E\right)$. If the current jetting would be the physical mechanism responsible for the observed negative ${\mathrm{MR}}_{\|}$ for samples S1, S2 and S3, a similar result would have been observed for S4, where the flake thickness is identical to the one of the other three samples. Thus, it is concluded that the origin of the observed negative ${\mathrm{MR}}_{\|}$ is due to the chiral anomaly in the WSM-II, WTe${}_{2}$.

Moreover, two test samples are also studied, in which the metal contacts are fabricated on the exfoliated flakes by employing electron beam lithography (EBL). For both samples, the resistance is found to be ∼$10^{5}\;\Omega$ and the samples display electronic properties befitting of a semiconductor. Such a change in the electronic behaviour in samples with contacts fabricated on flakes via EBL in comparison to the ones where the flakes are transferred onto the contacts, indicates that the exposure to chemicals and electron beams is detrimental for the semimetallic WTe${}_{2}$ flakes considered in this work. Moreover, both Au and Pt are found to provide robust Ohmic contacts to thin exfoliated WTe${}_{2}$ flakes.

### 3.4. Static Optical Reflectivity

Static reflectivity measurements as a function of *T*, $\mu_{0}H_{⊥}$ and polarization of the incident light $P_{L}$ have been performed on the exfoliated flakes. Optical reflectivity measured on WTe${}_{2}$ in the far infra-red (IR) and in the mid IR energy range was reported to point at charge compensation from electron and hole pockets at the Fermi level [72,73,74]. Theoretical calculations based on abinitio density functional theory have predicted anisotropic optical reflectivity in WTe${}_{2}$ for an energy range 0.5eV≤ℏω≤3.5eV as a function of the polarization [75]. Here, the experiments are carried out using a wide spectral range ultrafast pump-probe magneto-optical spectrometer at low-*T*/high-$\mu_{0}H$[76]. In particular, the probe beam of this set-up is sent through an additional element for the generation of supercontinuum pulses to perform reflectivity measurements. The details of the experimental set-up are provided in the Supplementary Materials and the experimental arrangement is shown in Figure S1. The reflectivity spectra are recorded on a ∼45 nm thick exfoliated WTe${}_{2}$ flake transferred onto a rigid SiO${}_{2}$/p${}^{++}$-Si substrate provided with markers to facilitate the identification of the flake under a microscope. The spectra are collected in the wavelength range between 450 nm and 700 nm at T=5K and at T=300K for $\mu_{0}H_{⊥}=0\;T$ and for $\mu_{0}H_{⊥}=3\;T$ and as a function of $P_{L}$, where the linear polarization of the supercontinuum pulses is rotated within the sample plane.

**Figure 9:**
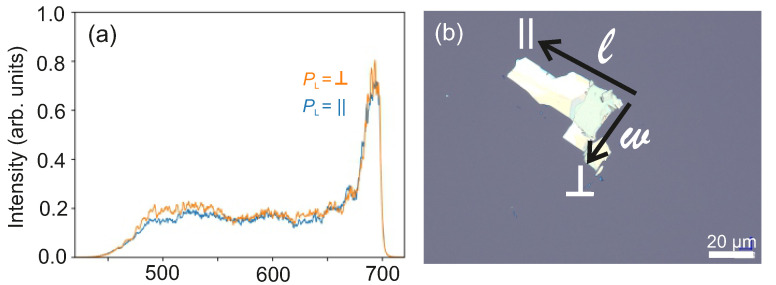
(**a**): Reference spectra of the supercontinuum pulses recorded for two crossed polarizations. The peak at ∼675 nm is associated with the seed pulse used for supercontinuum generation. (**b**): Optical microscopy image of the ∼45 nm $T_{d}$-WTe${}_{2}$ sample. The directions of the linear polarization used in the experiments (‖ and ⊥ to the flake long axis) are also indicated in the image.

**Figure 10:**
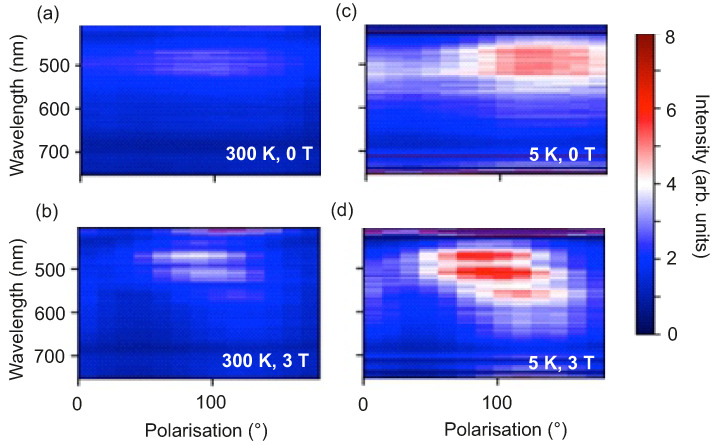
Reflectivity spectra for different *T* and $\mu_{0}H_{⊥}$ as a function of the polarization for (**a**): T=300K, $\mu_{0}H_{⊥}=0\;T$; (**b**): T=300K, $\mu_{0}H_{⊥}=3\;T$; (**c**): T=50K, $\mu_{0}H_{⊥}=0\;T$ and (**d**): T=5K, $\mu_{0}H_{⊥}=3\;T$.

The spectrum of the generated supercontinuum pulses, measured after passing the beam through a color filter and a polarizer, is shown in Figure 9a for two perpendicular linear polarization directions corresponding to the electric field of light $E_{L}$, defined as: (i) $P_{L}=120^{\circ }\equiv \;\|$ and (ii) $P_{L}=30^{\circ }\equiv \;⊥$, such that $E_{L}$ is respectively parallel and perpendicular to the long axis *l* of the measured WTe${}_{2}$ flake, as shown in Figure 9b. The spectrum spans the spectral range between 450 nm to 700 nm and does not depend on the direction of the linear polarization. a detailed investigation (with longer acquisition time to increase the signal-to-noise ratio) for the two polarizations $P_{L}=\|$ and $P_{L}=⊥$ is performed and the results are presented in Figure S2 of the Supplementary Materials. The measured reflectivity spectra are normalized with respect to a reference spectrum recorded by diverting the laser beam before the sample and are reported in Figure 10a–d as a function of the polarization angle, of *T*, and of $\mu_{0}H_{⊥}$. The spectra are shown as 2-dimensional (2D) maps, where the intensity is encoded in color scale. As discussed in detail in the Supplementary Materials and shown in Figure S3, the reflectivity spectra recorded both at T=300K and T=5K show a dependence on $P_{L}$, that is more pronounced for $\mu_{0}H_{⊥}=3\;T$ than for $\mu_{0}H_{⊥}=0\;T$. This anisotropic behavior is in agreement with what observed in the magnetotransport studies. Moreover, a comparison of the spectra recorded at a fixed *T* reveals that the changes in the spectra upon application of $\mu_{0}H_{⊥}=3\;T$ are more evident for $P_{L}=\|$ then for $P_{L}=⊥$. This behavior is elucidated quantitatively by evaluating the anisotropy *A* defined as:

(6) $$A=\frac{R(3\;T)-R(0\;T)}{R(0\;T)}$$

where R(3T) and R(0T) are the reflectivities measured for fixed light polarization and constant *T* in the presence and in the absence of magnetic field, respectively. As expected, the asymmetry is significantly pronounced in the data recorded for parallel light polarization. Thus, the magneto-optical reflectivity measurements complement the magnetotransport results and support the existence of anisotropic features in the electronic structure responsible for the observed out-of-plane electronic properties of the thin $T_{d}$-WTe${}_{2}$ flakes.

## 4. Conclusions

In summary, $T_{d}$-WTe${}_{2}$ flakes of thickness ∼45 nm have been fabricated via mechanical exfoliation and transferred onto rigid SiO${}_{2}$/*p*${}^{++}$-Si substrates with prepatterned electrical contacts using a viscoelastic dry transfer technique. The flakes are found to be stable in air and no chemical degradation is observed over an aging period of seven days. The two exemplary samples reported–S1 and S2–are fabricated using 10 nm thick Pt metal pads as Ohmic contacts in a van der Pauw geometry and designed by photolithography. In S1, the relative orientation of the $T_{d}$-WTe${}_{2}$ flake w.r.t. the four contact pads leads to an exact alignment of the directions of the applied electric field *E* and of the flake width *w*. In S2 the relative positions of the flake and the four Pt contacts result in a slight misalignment between *E* and *w*. Raman spectroscopic measurements carried out at room temperature reveal five Raman active modes, matching the theoretical predictions. The samples exhibit a large ${\mathrm{MR}}_{⊥}$ as high as 1200% at T=5K and for a $\mu_{0}H_{⊥}=7\;T$ applied along the c−axis. A $\mu_{0}H_{⊥}$-dependent turn-on $T_{\mathrm{Trans}}$ is observed, below which the samples undergo a MIT originating from the anisotropy of the Fermi surface. Both samples follow a Fermi liquid behavior for T≤50K. The anisotropy of the Fermi surface – in combination with the presence of electron and hole pockets in the electronic band structure leading to charge compensation – is concluded to be at the origin of the large positive ${\mathrm{MR}}_{⊥}$. The calculated $\mu_{\mathrm{av}}$∼5000 cm${}^{2}$V${}^{-1}$s${}^{-1}$ at T=5K for S1 is a property of the Fermi liquid, while for T≥50K the carrier mobility monotonically decreases due to the dominant electron-phonon coupling. The observed negative ${\mathrm{MR}}_{\|}$ for $\left[\mu_{0}H_{\|}\;\|\;\left(E\;\|\;w\right)\right]$ is a signature of chiral anomaly in $T_{d}$-WTe${}_{2}$ and is found to be remarkably sensitive to the relative orientation of the a− and b−axes w.r.t. the applied fields $\mu_{0}H_{\|}$ and *E*. The observed chiral anomaly persists up to T∼120 K, a temperature at least four times higher than the one previously reported for WTe${}_{2}$. A third sample S3 is also studied, which confirms the absence of current jetting, thereby allowing the conclusion that the quantum chiral anomaly is the origin of the observed negative ${\mathrm{MR}}_{\|}$. The anisotropic behavior of the studied WSM-II system is confirmed by studying the optical reflectivity of the flakes as a function of *T*, $\mu_{0}H$ and polarization of $E_{L}$ in the visible range of the electromagnetic spectrum. It is also concluded, that the Weyl semimetallic properties of exfoliated thin flakes of WTe${}_{2}$ are best observed when the flakes are transferred onto prefabricated metal Ohmic contacts, rather than when contacts are processed onto the flakes via EBL. The tunability of the large positive ${\mathrm{MR}}_{⊥}$ and the chiral anomaly-driven negative ${\mathrm{MR}}_{\|}$ as a function of the crystal axes and thickness, in combination with the chemical stability, pave the way for the application of 2D WSM-II WTe${}_{2}$ in the future generation of chiral electronic devices like, e.g., chiral batteries, and as active elements for the detection of ultraweak magnetic fields [77].

## Figures and Tables

**Table 1:** Estimated values of ${\mathrm{MR}}_{⊥}$, ${\mathrm{MR}}_{\|}$ and $\mu_{\mathrm{av}}$ at T = 5K and the critical temperature for observation of chiral anomaly in samples S1 and S2.

	${\mathrm{MR}}_{⊥}$(%); T = 5K; $\theta \;=\;90^{\circ }$	${\mathrm{MR}}_{\|}$(%); T = 5K; $\psi \;=\;90^{\circ }$	${\mathrm{MR}}_{\|}$(%); T = 5K; $\psi \;=\;0^{\circ }$	$\mu_{\mathrm{av}}$$\left({\mathrm{cm}}^{2}/V.s\right)$; T = 5K	Critical *T* (K)
S1	1200	36	−18	5000	120
S2	800	27	−5	4100	80

## Data Availability

The data presented in this study are available on request from the corresponding author.

## Supplementary Materials

The following are available online at https://www.mdpi.com/article/10.3390/nano11102755/s1, Figure S1: Schematic illustration of the set-up used for the reflectivity measurements. Figure S2: Reflectivity measured at the same temperature and magnetic field for $P_{L}=\|$ and $P_{L}=⊥$ for (a) 300 K, 0 T; (b) 300 K, 3 T: (c) 5 K, 0 T; (d) 5 K, 3 T. (e) Reflectivity measured at T=300K and T=5K for $\mu_{0}H_{⊥}=0\;T$ and $\mu_{0}H_{⊥}=3\;T$ for (e) $P_{L}=\|$ and (f) $P_{L}=⊥$. Figure S3: Asymmetry in the reflectivity as a function of wavelength for (a) $P_{L}=⊥$ and (b) $P_{L}=\|$. Figure S4: Optical image of the sample S4. Figure S5: (a) $R_{\mathrm{xx}}$ as a function of *T* at $\mu_{0}H_{⊥}=0$ and $\mu_{0}H_{⊥}\neq 0$: (b) ${\mathrm{MR}}_{⊥}$ as a function of $\mu_{0}H_{⊥}$ measured in the range 5K≤T≤150K; (c) ${\mathrm{MR}}_{⊥}$ measured as a function of $\mu_{0}H_{⊥}$ at $\theta =0^{\circ }$, $\theta =45^{\circ }$ and $\theta =90^{\circ }$ at T=5K; (d) FC ${\mathrm{MR}}_{⊥}$ as a function of *T* recorded for $\mu_{0}H_{⊥}=3\;T$ and 7T; (e) Normalized ${\mathrm{MR}}_{⊥}$ defined as the ratio of ${\mathrm{MR}}_{⊥}\left(T\right)$ to ${\mathrm{MR}}_{⊥}\left(5\;K\right)$ recorded by applying $\mu_{0}H_{⊥}=3\;T$ and $\mu_{0}H_{⊥}=7\;T$; (f) Calculated Kohler’s plots for the range 5K≤T≤100K for S4. Figure S6: (a) Zero field cooled $R_{\mathrm{xx}}$ as a function of *T* with $\mu_{0}H_{⊥}=0$ for the sample S4. The experimental data is represented by the solid line, while the dashed line is a Fermi liquid theory fitting curve; (b) ${\mathrm{MR}}_{\|}$ recorded as a function of $\mu_{0}H_{\|}$ at T=5K for $\psi =0^{\circ }$ and for $\psi =90^{\circ }$ for S4.

## Abbreviations

The following abbreviations are used in this manuscript:

MR	Magnetoresistance
WSM	Weyl semimetals
WQP	Weyl quasiparticles
ABJ	Adler–Bell–Jakiw
TMDC	Transition metal dichalcogenide
AFM	Atomic force microscopy
PDMS	Polydimethylsiloxane
LIA	Lock-in amplifier
ZFC	Zero field cooled
FC	Field cooled
MIT	Metal-to-insulator transition
NLMR	Negative longitudinal magnetoresistance
LLL	Lowest Landau level
EBL	Electron beam lithography
IR	Infra-red
