# Supplementary material for: Positive Magnetoresistance and Chiral Anomaly in Exfoliated Type-II Weyl Semimetal Td-WTe2
Source: Nanomaterials (Basel). 2021 Oct 18;11(10):2755. doi: 10.3390/nano11102755 (PMC8541530; doi:10.3390/nano11102755)
Supplement: Supplementary file 1 [file nanomaterials-11-02755-s001.zip › nanomaterials-1373068-supplementary.pdf]

nanomaterials

MDPI

Article

Positive Magnetoresistance and Chiral Anomaly in Exfoliated Type-II Weyl Semimetal T_d-WTe_2

Rajdeep Adhikari [1,*,†], Soma Adhikari [1,†], Bogdan Faina [1], Mark Terschanski [2], Sophie Bork [2], Claudia Leimhofer [3], Mirko Cinchetti [2] and Alberta Bonanni [1,*]

1 Institut für Halbleiter-und-Festkörperphysik, Johannes Kepler University, Altenbergerstr. 69, A-4040 Linz, Austria; soma.adhikari@jku.at (S.A.); Bogdan.Faina@jku.at (B.F.)
2 Department of Physics, TU Dortmund, Otto-Hahn-Straße 4, 44227 Dortmund, Germany; marc.terschanski@tu-dortmund.de (M.T.); sophie.bork@tu-dortmund.de (S.B.); mirko.cinchetti@tu-dortmund.de (M.C.)
3 Institut für Polymerwissenschaften, Johannes Kepler University, Altenbergerstr. 69, A-4040 Linz, Austria; claudia.leimhofer_1@jku.at
* Correspondence: Rajdeep.Adhikari@jku.at (R.A.); Alberta.Bonanni@jku.at (A.B.); Tel.: +43-732-2468-9664 (A.B.)
† These authors contributed equally to this work.

check for updates

Citation: Adhikari, R.; Adhikari, S.; Faina, B.; Terschanski, M.; Bork, S.; Leimhofer, C.; Cinchetti, M.; Bonanni, A. Positive Magnetoresistance and Chiral Anomaly in Exfoliated Type-II Weyl Semimetal T_d-WTe_2. *Nanomaterials* **2021**, *11*, 2755. https://doi.org/10.3390/nano11102755

Academic Editor: Yann-Wen Lan, Der-Hsien Lien

Received: 24 August 2021
Accepted: 10 October 2021
Published: 18 October 2021

Publisher's Note: MDPI stays neutral with regard to jurisdictional claims in published maps and institutional affiliations.

Copyright: © 2021 by the authors. Licensee MDPI, Basel, Switzerland. This article is an open access article distributed under the terms and conditions of the Creative Commons Attribution (CC BY) license (https://creativecommons.org/licenses/by/4.0/).

1. Optical Studies: Reflectivity Measurements with Supercontinuum

Static reflectivity measurements as a function of temperature T, applied magnetic field $\mu_0 H$ and polarization of the incident light P_L have been performed using a recently developed wide spectral range ultrafast pump-probe magneto-optical spectrometer at low T, high-$\mu_0 H$ and electric fields E [1]. Here, only the probe beam of the set-up equipped with an additional element for the generation of supercontinuum pulses is used. The detection of the reflectivity signal is carried out using a detector assembly with high spectral resolution, sketched in Figure S1. In particular, only 35% of the laser beam generated by a ytterbium-based amplified laser system (Pharos, Light Conversion) with a central wavelength of 1028 nm, 20 W total power and 200 kHz repetition rate has been employed. The pulse with a power of 7 W is fed into an optical parametric amplifier (OPA) to generate a seed pulse at 750 nm, which is then send through a polarizer and focused on a sapphire crystal to generate broad and stable supercontinuum pulses. Finally, the seed pulse is filtered out using a 700 nm short-pass color filter, polarized by a $\lambda/2$ plate and focused onto the sample using a microscope consisting of components with very low magnetic susceptibility.

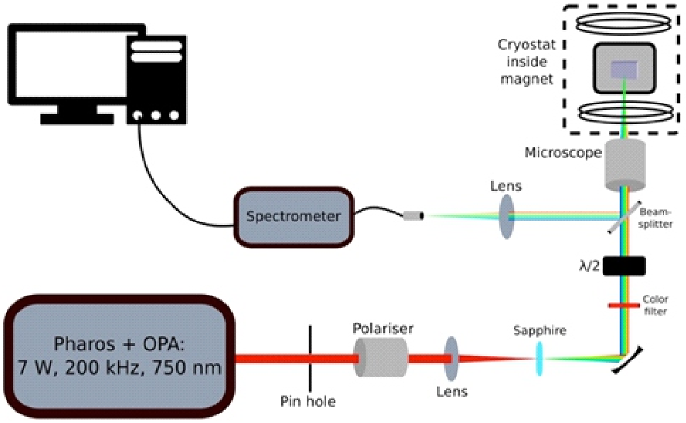

Figure S1. Schematic illustration of the set-up used for the reflectivity measurements.

The spectrum of the generated supercontinuum pulses is collected after the color filter and the polarizer. Two states of polarization of the incident light are used defined as: (i)

$P_L = 120°$ and (ii)$P_L = 30°$, such that E_L is respectively parallel and perpendicular to the long axis of the measured WTe_2 flake, as shown in Fig. 8(b) in the main text. The recorded spectra for the two polarizations are shown in Fig. 8(a) while the relative orientation of P_L w.r.t. the long axis of the T_d-WTe_2 flake is reported in Fig. 8(b) of the main text. The spectrum is recorded over the spectral range between 450 nm and 700 nm. The sample is mounted inside a flow cryostat with a low temperature limit $T = 5\,K$ and then moved into the bore of a magnet to apply magnetic fields up to $\mu_0 H = 9\,T$. Since here the reflection of the light from the flakes is measured, the microscope is also employed to collect the laser beam reflected from the sample. A beam-splitter directs the signal onto the Thorlabs spectrometer CCS200/M which is then digitized by a computer.

As discussed in Fig. 9 of the main text, an anisotropic behavior is observed in the reflectivity spectra of the studied flake as a function of T and P_L. In order to understand this behavior, a detailed investigation (with longer acquisition time to increase the signal-to-noise ratio) for the two polarizations $P_L =\parallel$ and $P_L =\perp$ is performed. In addition to the previous experimental protocol, the spectrum recorded from the substrate has been substracted for each measurement settings before normalizing it to the reference spectrum. The data are shown in Figure S2.

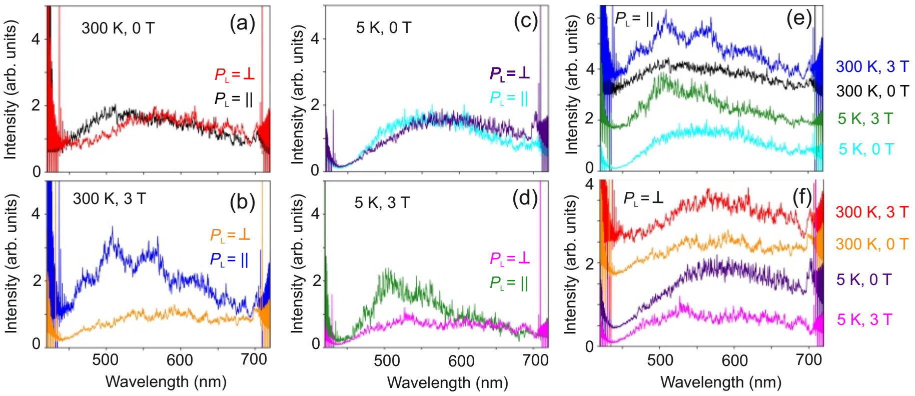

Figure S2. Reflectivity measured at the same temperature and magnetic field for $P_L =\parallel$ and $P_L =\perp$ for (**a**) 300 K, 0 T; (**b**) 300 K, 3 T: (**c**) 5 K, 0 T; (**d**) 5 K, 3 T. (**e**) Reflectivity measured at $T = 300\,K$ and $T = 5\,K$ for $\mu_0 H_\perp = 0\,T$ and $\mu_0 H_\perp = 3\,T$ for (**e**) $P_L =\parallel$ and (f) $P_L =\perp$.

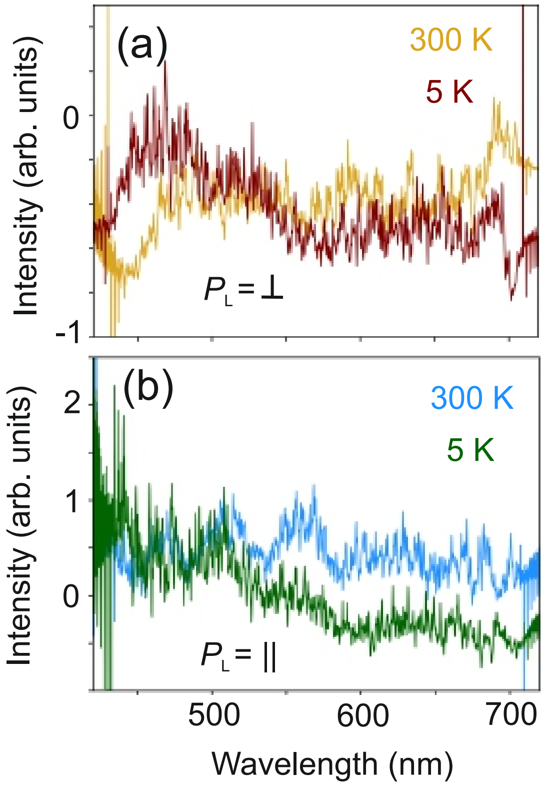

Figure S3. Asymmetry in the reflectivity as a function of wavelength for (**a**) $P_L = \perp$ and (**b**) $P_L = \|$.

As shown in Figure S2(a) and in Figure S2(c) at $T = 300$ K and at $T = 5$ K, respectively, no anisotropy in the reflectivity spectra is observed for $P_L = \perp$ and $P_L = \|$ when $\mu_0 H_\perp = 0$ T. However, upon application of a magnetic field $\mu_0 H_\perp = 3$ T, an anisotropy in the recorded reflectivity of the T_d-WTe_2 flake is detected both at $T = 300$ K and at $T = 5$ K, as reported in Figure S2(b) and Figure S2(d), respectively. The asymmetry defined by Eq. (5) in the main text is shown as a function of wavelength in Figure S3. The asymmetry is found to be pronounced in the data recorded for $P_L = \|$, and prominent features at wavelengths of 475 nm, 505 nm and 560 nm are observed.

2. Magnetotransport Properties: Gold Contact

For comparison, a S4 sample has been fabricated using Au (instead of Pt) as contact metal. Due to the poor adhesion of Au to SiO_2, a 10 nm Ti adhesion layer is first deposited, followed by a 40 nm thick Au layer. The Ti/Au metal stack is deposited *via* e-beam evaporation at a base pressure of (1×10^{-6}) mbar in a Belzar e-beam evaporation system. A thin WTe_2 flake of thickness ∼45 nm is then transferred onto the prepatterned Ti/Au metal contacts using the deterministic viscoelastic transfer technique as discussed in the main text. The optical image of the complete sample is shown in Figure S4. The contacts C_{41}, C_{42}, C_{43} and C_{44} are employed to measure the magnetotransport properties of the flake in a 4-probe measuring geometry. Here, the excitation current I_{ac} is applied between C_{41} and C_{42} while the voltage V_{xx} is recorded across C_{43} and C_{44}. Hence, the electric field E due to I_{ac} is highly misaligned w.r.t. w, as reported in the optical microscopy image of S4 given in Figure S4.

The evolution of R_{xx} as a function of T both in the presence and in the absence of $\mu_0 H_\perp$ in the interval $5\,\mathrm{K} \leq T \leq 250\,\mathrm{K}$ is shown in Figure S5a. It is found, that the $R_{xx} - T$ curve for sample S4 is similar to those discussed in the main text for S1 and S2. The turn-on transition temperature T_{Trans} as a function of $\mu_0 H_\perp$ is comparable to the one estimated in S1 and S2. The evolution of $MR_\perp$ as a function of $\mu_0 H_\perp$, T and θ is reported in Figure S5b,c respectively. The large unsaturating positive and anisotropic $MR_\perp$ recorded for S4 indicates

that the contacting metal has no significant effect on the electronic properties of the thin WTe_2 flakes.

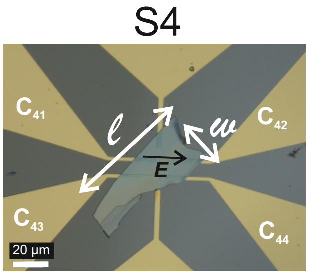

Figure S4. Optical image of the sample S4.

The $MR_\perp$ as a function of T measured for an applied $\mu_0 H = 3\,T$ and for $\mu_0 H = 7\,T$ are reported in Figure S5d, while the normalized $MR_\perp$ is presented Figure S5e, respectively. The observed behavior of $MR_\perp$ matches the ones established for samples S1 and S2 while the deviation of the electronic properties of S4 from the Kohler's rule, as presented in Figure S5f, is a proof of the charge compensation mechanism between the electron and hole pockets at the tilted Fermi surface of WTe_2, typical of WSM-II systems.

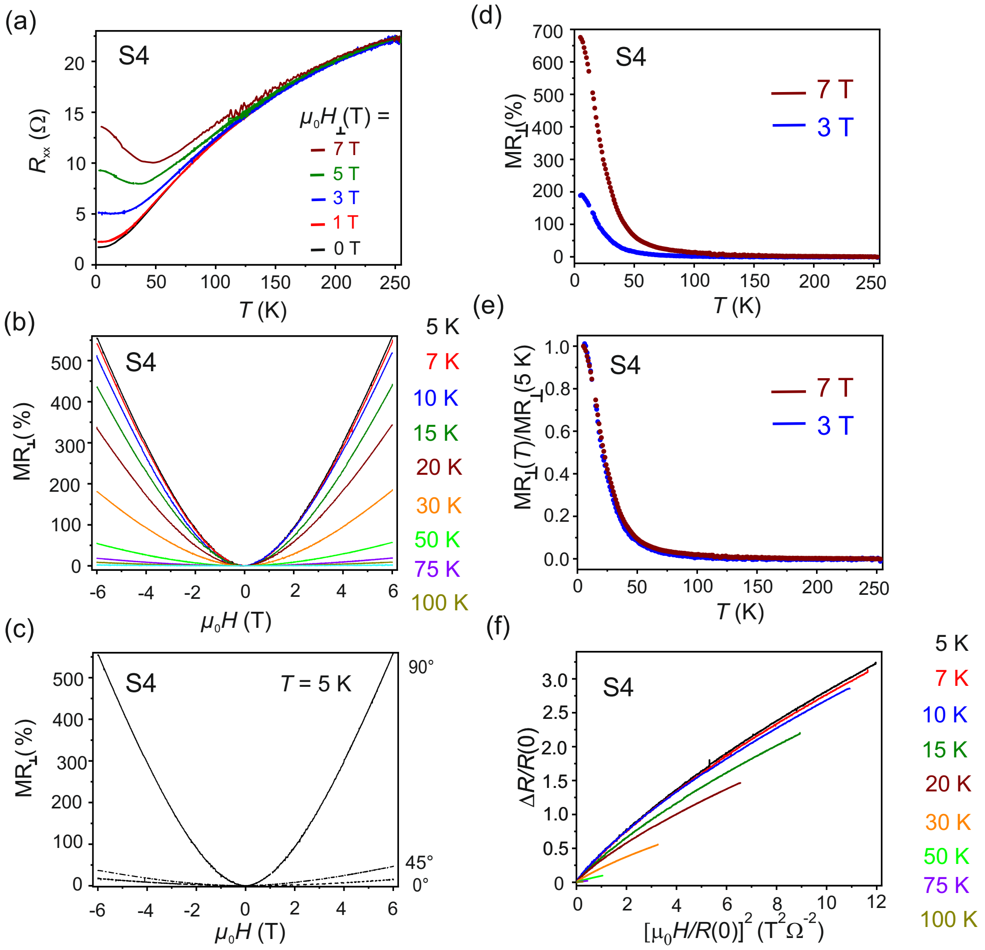

Figure S5. (**a**) R_{xx} as a function of T at $\mu_0 H_\perp = 0$ and $\mu_0 H_\perp \neq 0$: (**b**) $MR_\perp$ as a function of $\mu_0 H_\perp$ measured in the range $5\,K \leq T \leq 150\,K$; (**c**) $MR_\perp$ measured as a function of $\mu_0 H_\perp$ at $\theta = 0°$, $\theta = 45°$ and $\theta = 90°$ at $T = 5\,K$; (**d**) FC $MR_\perp$ as a function of T recorded for $\mu_0 H_\perp = 3\,T$ and $7\,T$; (**e**) Normalized $MR_\perp$ defined as the ratio of $MR_\perp(T)$ to $MR_\perp(5\,K)$ recorded by applying $\mu_0 H_\perp = 3\,T$ and $\mu_0 H_\perp = 7\,T$; (**f**) Calculated Kohler's plots for the range $5\,K \leq T \leq 100\,K$ for S4.

The evolution of R_{xx} as a function of T for S4 follows a Fermi liquid behavior and is shown in Figure S6a. This behavior is consistent with the one of the samples S1 and S2. However, no chiral anomaly is observed for S4, as seen in the bahavior of $MR_{\|}$ as a function of $\mu_0 H_{\|}$ and ψ as presented in Figure S6b. The absence of negative $MR_{\|}$ for $\psi = 0°$ indicates that, in contrast to the case of S1 and S2, the w of S4 is not aligned along the $b-$axis of the orthorhombic lattice resulting in the positive $MR_{\|}$. It is concluded, that though the metal used for the Ohmic contact and the air aging have negligible effect on the electronic behavior of the WTe_2 flakes reported here, the relative orientation of the flakes w.r.t. E, b and $\mu_0 H_{\|}$ plays a major role in the observation of the chiral anomaly.

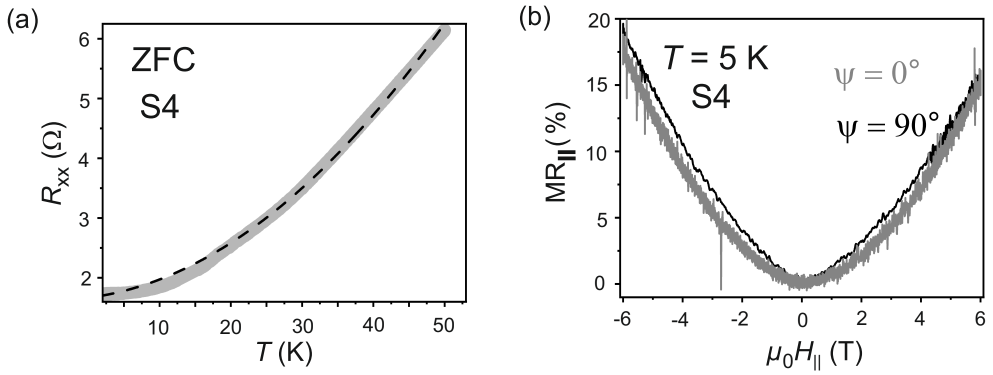

Figure S6. (**a**) Zero field cooled R_{xx} as a function of T with $\mu_0 H_{\perp} = 0$ for the sample S4. The experimental data is represented by the solid line, while the dashed line is a Fermi liquid theory fitting curve; (**b**) $MR_{\|}$ recorded as a function of $\mu_0 H_{\|}$ at $T = 5$ K for $\psi = 0°$ and for $\psi = 90°$ for S4.

References

1. F. Mertens, M. Terschanski, D. Mönkebüscher, S. Ponzoni, D. Bossini, and M. Cinchetti, "Wide spectral range ultrafast pump–probe magneto-optical spectrometer at low temperature, high-magnetic and electric fields," Rev. Sci. Instrum. **91**, 113001 (2020)
